# More than just a simple barrier

**DOI:** 10.7554/eLife.97247

**Published:** 2024-03-27

**Authors:** Erandi Velazquez-Miranda, Ming He

**Affiliations:** 1 Department of Pathology, Heersink School of Medicine, The University of Alabama at Birmingham Birmingham United States; 2 Division of Molecular and Cellular Pathology, Department of Pathology, The University of Alabama Birmingham United States

**Keywords:** endothelial cells, ERG, inflammation, atherosclerosis, vascular biology, epigenetics, Human

## Abstract

Endothelial cell subpopulations are characterized by unique gene expression profiles, epigenetic landscapes and functional properties.

**Related research article** Adelus ML, Ding J, Tran BT, Conklin AC, Golebiewski AK, Stolze LK, Whalen MB, Cusanovich DA, Romanoski CE. 2023. Single cell ‘omic profiles of human aortic endothelial cells in vitro and human atherosclerotic lesions ex vivo reveals heterogeneity of endothelial subtype and response to activating perturbations. *eLife* **12**:RP91729. doi: 10.7554/eLife.91729.

Our blood vessels are lined with a single layer of endothelial cells, known as the vascular endothelium, which provides a permeable barrier to protect the blood from toxins and other harmful substances. Endothelial cells have many important roles, including regulating the contraction and relaxation of the blood vessels, enabling exchange of molecules between blood and tissues, and regulating immune response and inflammation.

Advances in cell biology and molecular techniques have shown that the endothelium is more than just a uniform layer lining the blood vessels. Endothelial cells vary greatly among different tissues and even between individual cells within a region (Kalucka et al., 2020; Paik et al., 2020; Kalluri et al., 2019; Chen et al., 2024). These differences, which can include gene expression, epigenetic landscape, functional properties and responsiveness to stimuli, suggest distinct and specialized roles in vascular physiology and pathology (Figure 1).

**Figure 1.**
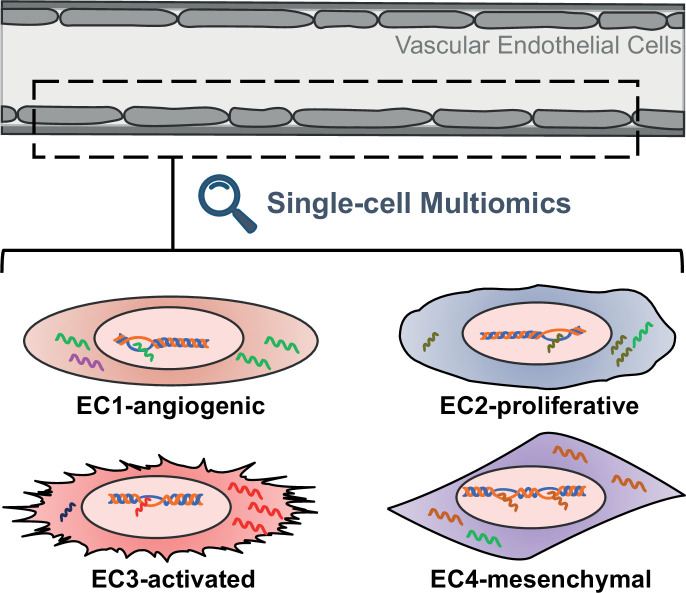
Schematic representing the different properties of endothelial cell subtypes. The cells of the vascular endothelium are extremely heterogenous, which could affect their role in vascular function and disease. EC1 cells are involved in the formation of new blood vessels, EC2 cells have a fast cell turnover, and both EC3 cells and EC4 cells show mesenchymal properties.

Disrupted endothelial function has been linked to atherosclerosis, a thickening of the arteries caused by a build-up of plaque in the endothelium, which can lead to ischemic heart diseases. Many of the endothelial cells in atherosclerotic plaques no longer express typical endothelial cell markers and instead become more like another type of cell, called a mesenchymal cell, which acquires properties or releases molecules that overactivate the immune system, worsening the disease. Understanding the extent of the heterogeneity of endothelial cells promises to yield valuable insights into vascular biology and could provide new therapeutic targets for cardiovascular diseases. However, studies that quantify the heterogeneity of endothelial cells in live animals and cell models are lacking. Now, in eLife, Casey Romanoski and colleagues at the University of Arizona – including Maria Adelus as first author – report a comprehensive analysis of endothelial cell diversity and their unique responses under atherosclerosis-like conditions (Adelus et al., 2023).

To delve into the intricacies of endothelial cells within human aortas, the team combined experiments with human aortic cells grown in the laboratory and analysis of publicly available single-cell gene expression data from human arterial specimens. This uncovered four distinct subtypes of endothelial cells, which are characterized by unique gene expression profiles, epigenetic landscapes and functional properties. For example, the expression profile of EC1 cells suggests they are involved in the formation of new blood vessels, while EC2 cells display rapid growth characteristics. On the other hand, both EC3 and EC4 cells resemble mesenchymal cells, suggesting they have transitioned from their original cell type.

Next, Adelus et al. investigated how these different populations respond to factors known to be important for endothelial cell biology and the development of atherosclerosis. The experiments unveiled distinct reactions across endothelial populations. The EC1 and EC2 populations, which mostly represent healthy endothelial cells, were most responsive when a transcription factor required for endothelial cell specification and homeostasis was deactivated. In contrast, EC3 populations were most affected by stimulation with a cytokine to mimic inflammation, whereas EC4s reacted to activation of a specific growth factor that allows cells to transition to different cell types. Furthermore, Adelus et al. identified accessible chromatin regions unique to EC2 and EC4 populations that are enriched with genetic variations associated with coronary artery disease. This suggests that EC2 and EC4 populations could be involved in mechanisms underlying the development and progression of coronary artery disease.

Taken together, these findings offer valuable insights into the underlying pathophysiology of atherosclerosis and suggest that the characteristics of individual endothelial cells enable them to respond uniquely to various pathological processes, potentially playing distinct roles in the progression of vascular diseases. The identification of heterogeneous endothelial subtypes underscores the complexity of endothelial cell populations and emphasizes the necessity for further investigation into their roles in both health and disease. By integrating laboratory-based experiments with publicly available datasets, Adelus et al. enhanced the reliability of the findings and established a solid foundation for future research endeavors.

Thus far, single-cell studies have paved the way for unraveling the diversity of vascular endothelial biology. For future studies, it will be crucial to standardize how different populations of endothelial cells are characterized and identified to enable more consistent evaluations based on uniform criteria. A next step would be to profile gene expression and epigenetic landscapes of the endothelial cell clusters to define their unique roles and to distinguish athero-protective from athero-prone cells. Once the latter are identified, more accurate cell-targeting strategies, such as nanoparticles, can be developed to ultimately improve the treatment of cardiovascular diseases with limited side effects.
